# Melatonin: a promising therapy to combat type 2 airway inflammation via MT1-Sirt1 pathway

**DOI:** 10.3389/fphar.2026.1805178

**Published:** 2026-04-17

**Authors:** Zhe Zhang, Jie Jiang, Guilian Chen, Xiuqin Zhang, Jipeng Wang, Baolan Wang

**Affiliations:** 1 Physical Examination Center, The Affiliated Huai’an No.1 People’s Hospital of Nanjing Medical University, Huai’an, Jiangsu, China; 2 Department of Respiratory Medicine, The Affiliated Huai’an No.1 People’s Hospital of Nanjing Medical University, Huai’an, Jiangsu, China; 3 Department of Respiration Medicine, The First Affiliated Hospital of Soochow University, Suzhou, Jiangsu, China

**Keywords:** circadian genes, epithelial-mesenchymal transition, melatonin, MT1-Sirt1, type 2 airway inflammation

## Abstract

**Backgrounds:**

Type 2 asthma is characterized by airway inflammation, mucus hypersecretion, and remodeling, and circadian rhythm dysregulation is implicated in its pathogenesis. Melatonin, a key circadian hormone, modulates inflammatory signaling, but its role in type 2 airway inflammation remains unclear. This study investigated whether melatonin alleviates airway inflammation and epithelial-mesenchymal transition (EMT) through the melatonin receptor 1 (MT1)-Sirtuin 1 (Sirt1) signaling pathway and circadian clock regulation.

**Methods:**

An ovalbumin (OVA)-induced mouse model of type 2 airway inflammation and cultured airway epithelial cells were used. Lung structural remodeling and mucus production were evaluated using hematoxylin and eosin, Masson’s trichrome, and periodic acid–Schiff staining. Airway inflammation was assessed by differential inflammatory cell counts in bronchoalveolar lavage fluid. The mRNA and protein expression of circadian clock genes (CRY1 and PER1) and key components of the MT1–Sirt1 pathway were assessed by quantitative real-time PCR, Western blotting, and immunohistochemistry. EMT-related markers were further examined to explore downstream mechanisms.

**Results:**

Melatonin treatment activated MT1-Sirt1 signaling and reduced the expression of circadian clock genes CRY1 and PER1. These effects were accompanied by decreased airway inflammation, reduced mucus production, and attenuation of epithelial-mesenchymal transition (EMT) in airway epithelial cells, all of which reached statistical significance.

**Conclusion:**

Our findings identify a novel MT1–Sirt1–circadian gene regulatory axis through which melatonin mitigates type 2 airway inflammation and airway remodeling. These results highlight the therapeutic potential of melatonin for type 2 asthma. Limitations include the use of a single animal model and analysis at a single time point.

## Graphical Abstract

**Figure:**
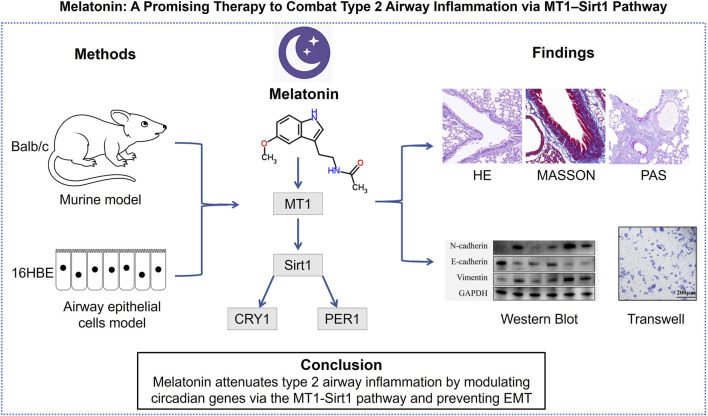

## 1 Introduction

Melatonin is best known as a hormone that regulates circadian rhythms; however, accumulating evidence indicates that it also possesses anti-inflammatory, antioxidant, and immunomodulatory properties (Campos et al., 2023; Kim et al., 2023; Posadas et al., 2012). In addition to its well-known role in circadian rhythm maintenance, melatonin has been increasingly recognized for its anti-inflammatory, antioxidant, and immunomodulatory properties. Type 2 airway inflammation is a key pathological feature of asthma and several chronic respiratory diseases and is characterized by airway inflammation, mucus hypersecretion, and airway remodeling (Ragnoli et al., 2022; Chen et al., 2023). Given that these pathological changes significantly impair patients’ quality of life and place a substantial burden on healthcare systems (Wang et al., 2020), a better understanding of the regulatory mechanisms underlying type 2 airway inflammation is essential for the development of new therapeutic strategies.

Recent studies suggest that melatonin has immunoregulatory effects that may play a role in asthma pathogenesis (Tonon et al., 2021; Li et al., 2024; Liu et al., 2021). It has been shown to influence innate immune cell activity, reduce oxidative stress, and engage multiple signaling pathways (Kikuchi et al., 2022). In addition, melatonin can regulate the expression of core circadian clock genes, which may in turn influence airway inflammatory response (Pfeffer et al., 2018; Sundar et al., 2015; Durrington et al., 2018). Growing evidence indicates that disturbances in circadian rhythms can worsen inflammation and promote asthma progression, underscoring the close link between circadian regulation and immune function (Costello and Gumz, 2021).

Although research in this area continues to expand, the precise mechanisms through which melatonin regulates immune responses in airway inflammation remain incompletely understood. Previous studies have shown that melatonin can modulate immune responses and inflammatory processes in various pathological contexts, including through regulation of autophagy and cellular signaling pathways (Farez et al., 2015; Tao et al., 2018; Cesario et al., 2022). In addition, melatonin has been reported to attenuate airway inflammation through activation of the silent information regulator 1 (Sirt1) signaling pathway (Peng et al., 2018) and through interactions with its membrane receptors, MT1 and MT2 (Mao et al., 2021). However, the potential interaction between melatonin receptor signaling, Sirt1 activation, and circadian clock gene regulation in type 2 airway inflammation has not been fully elucidated. In particular, whether melatonin regulates circadian rhythm–related genes through the MT1–Sirt1 signaling pathway and how this mechanism contributes to airway inflammatory responses and epithelial–mesenchymal transition (EMT) remain unclear. This study therefore focuses on a previously underexplored regulatory axis linking MT1–Sirt1 signaling with circadian clock gene regulation in type 2 airway inflammation.

Therefore, this study investigated whether melatonin regulates circadian clock genes, particularly CRY1 and PER1, through the MT1-Sirt1 signaling pathway in type 2 airway inflammation. Using an ovalbumin (OVA)-induced mouse model and airway epithelial cells, we examined the effects of melatonin on airway inflammation, circadian gene expression, and epithelial-mesenchymal transition (EMT). Our findings provide insight into the interaction between circadian rhythm regulation and inflammatory signaling and suggest a potential therapeutic role for melatonin in asthma.

## 2 Materials and methods

### 2.1 Animals

Female BALB/c mice (8 weeks old, 18–20 g) were obtained from Shanghai BK/KY Biotechnology Co., Ltd. Female mice were used because they are more susceptible to allergen-induced type 2 airway inflammation and exhibit stronger Th2 immune responses (Takeda et al., 2013). Animals were maintained under controlled environmental conditions (20 °C–26 °C, 12 h light/dark cycle) with free access to standard chow and water. All animal procedures were reviewed and approved by the Animal Care and Use Committee of the Affiliated Huai’an No.1 People’s Hospital of Nanjing Medical University (Approval No. DW-P-2023–001–22).

### 2.2 OVA-induced allergic airway inflammation and drug treatment

Mice were randomly assigned to five experimental groups (n = 6 per group): Control, OVA model, and OVA combined with melatonin, Luzindole, 4-P-PDOT, EX527, or Resveratrol (Luzindole: a non-selective MT1/MT2 antagonist, 4-P-PDOT: a selective MT2 antagonist, EX527: a Sirt1 inhibitor, and resveratrol: a Sirt1 activator). Details of the chemicals and drugs are provided in Supplementary Table S3. To induce allergic airway inflammation, mice in the OVA-treated groups received intraperitoneal injections of an ovalbumin (OVA) sensitizing solution (0.05 mg/mL OVA with aluminum hydroxide in PBS) on days 0, 7, 14, and 21. From days 28–32, mice were challenged intranasally with 50 μL of 5% OVA in saline once daily. During the challenge period, mice received daily intraperitoneal injections of melatonin (15 mg/kg) (Zhang et al., 2024), Luzindole (30 mg/kg) (Lu et al., 2024), 4-P-PDOT (10 mg/kg) (Ren et al., 2024), EX527 (10 mg/kg) (Jiang et al., 2024), or Resveratrol (30 mg/kg) (Gu et al., 2019; Sener et al., 2006) at indicated doses. Control mice received equal volumes of vehicle. All animals were euthanized 24 h after the final OVA challenge (day 33). Researchers were not blinded to group assignment. The experimental protocol was conducted according to our previously published methodology (Wang et al., 2025).

### 2.3 Study outcomes definition

The primary outcome was the degree of airway inflammation, assessed by bronchoalveolar lavage fluid (BALF) cell counts. Secondary outcomes included: (1) mucus production (PAS staining); (2) lung structural remodeling (H&E and Masson’s trichrome staining); (3) expression of circadian clock genes (CRY1 and PER1); and (4) expression of MT1-Sirt1 pathway components. Exploratory outcomes included epithelial-mesenchymal transition (EMT)-related markers. All outcomes were assessed by blinded investigators where applicable.

### 2.4 Specimen processing

#### 2.4.1 Bronchoalveolar lavage fluid (BALF) collection and analysis

Twenty-four hours after the final challenge, mice were sacrificed between 09:00–10:00 AM. The left main bronchus was ligated, and bronchoalveolar lavage was performed on the right lung using ice-cold PBS (0.3 mL per wash, five washes). The lavage fluid recovered via tracheal cannula from three washes per mouse and pooled. and centrifuged at 1,000 rpm for 10 min at 4 °C with an average recovery rate >85% and no significant differences between groups. Cell pellets were resuspended in 200 μL PBS, and total leukocyte counts were determined using a hemocytometer. Differential cell counts were assessed on cytospin slides by an investigator blinded to group allocation.

#### 2.4.2 Serum collection and analysis

Peripheral blood samples were collected via retro-orbital puncture and allowed to clot at room temperature for 2 h. Serum was separated by centrifugation at 3,000 rpm for 15 min and stored at −20 °C until analysis. Serum levels of immunoglobulin E (IgE), MUC5AC, cortisol, and melatonin were measured using commercially available enzyme-linked immunosorbent assay (ELISA) kits. Concentrations of interleukin (IL)-4, IL-5, and IL-13 were also quantified according to the manufacturers’ instructions. All ELISA assays were performed in duplicate with standard curves (*R* ^2^ > 0.98). Intra-assay and inter-assay coefficients of variation were <10% for all kits. Details of the experimental procedures and reagents can be found in Supplementary Tables S3 and S4.

#### 2.4.3 Lung tissue processing

After blood sampling, mice were euthanized by cervical dislocation. Lungs were excised and rinsed with physiological saline. The left lung was fixed by immersion in 4% paraformaldehyde for histological analysis, whereas the right lung was snap-frozen in liquid nitrogen for 30 min and stored at −80 °C for subsequent molecular analyses.

#### 2.4.4 Lung tissue morphological analysis

Lung tissues fixed in 4% paraformaldehyde were gently agitated for 48 h, followed by routine dehydration, paraffin embedding, and sectioning at a thickness of 4–5 μm. Tissue sections were stained with hematoxylin and eosin (H&E) to evaluate general histopathological changes, Masson’s trichrome staining was used to assess collagen deposition and fibrosis, and periodic acid–Schiff (PAS) staining was performed to visualize mucus production. Ten non-overlapping fields per section (5-7 airways per field) were selected by systematic random sampling. Inflammation (H&E) and mucus (PAS) were scored 0–4, fibrosis (Masson) was quantified as % positive area. Histological alterations were digitally captured and quantified using ImageJ with standardized thresholds, and independently scored by two blinded pathologists using a predefined system; disagreements were resolved by consensus or a third pathologist.

#### 2.4.5 Immunohistochemical staining

Paraffin-embedded lung sections were incubated at 60 Covernight, then deparaffinized and rehydrated through graded ethanol solutions. Endogenous peroxidase activity was blocked using 3% hydrogen peroxide. Antigen retrieval was performed in citrate buffer under high-pressure conditions. Sections were subsequently blocked with 10% bovine serum albumin for 1 h at room temperature and incubated with primary antibodies overnight at 4 °C. After incubation with HRP-conjugated secondary antibodies, immunoreactive signals were developed using diaminobenzidine (DAB). Sections were counterstained with hematoxylin, imaged using a light microscope, and subsequently quantified using ImageJ software. Experimental procedures and reagents are summarized in Supplementary Tables S3 and S4.

#### 2.4.6 RNA extraction and qRT-PCR

Total RNA was isolated from lung tissues using a commercial RNA extraction kit under RNase-free conditions. RNA purity was assessed by measuring the A260/A280 ratio. For cDNA synthesis, 1 μg of total RNA was used for reverse transcription. Complementary DNA (cDNA) was synthesized using a reverse transcription kit incorporating genomic DNA removal. Quantitative real-time PCR (qRT-PCR) was performed using SYBR Green chemistry on a real-time PCR system. All reactions were performed in triplicate, with amplification efficiencies between 90% and 110% and *R* ^2^ > 0.98. Relative gene expression levels were calculated using the 2^−ΔΔCt^ method, and primer sequences are listed in Supplementary Table S1.

#### 2.4.7 Cell culture, transfection, and serum shock

Human bronchial epithelial 16HBE cells were maintained in RPMI-1640 medium supplemented with 10% fetal bovine serum and antibiotics at 37 °C in a humidified 5% CO_2_ atmosphere. Lentiviral particles targeting BMAL1 were produced in HEK293T cells by co-transfection of the shRNA transfer plasmid together with packaging plasmids using PEI. Viral supernatants were collected and used to infect 16HBE cells in the presence of polybrene (Lesch et al., 2011). Stable knockdown cells were selected with puromycin, and silencing efficiency was confirmed by qRT-PCR. Circadian synchronization was achieved by serum starvation followed by a 2-h pulse of 50% horse serum. ZT0 was defined as the time of serum shock washout, and subsequent ZT values indicate hours post-synchronization *in vitro*. Cells were harvested every 4 h from ZT0 to ZT44 for rhythmic gene expression analysis (Balsalobre et al., 1998). At each time point, the culture medium was aspirated, cells were rapidly washed with cold PBS, and immediately lysed on the plate using RNA extraction reagent.

#### 2.4.8 RNA extraction and qRT-PCR of cells

Total RNA from cultured cells was extracted and reverse-transcribed as described for lung tissues. Quantitative PCR analysis was conducted using identical reaction conditions. Primer sequences are provided in Supplementary Table S2.

#### 2.4.9 Transwell migration assay

Cell migratory capacity was assessed using Transwell chambers (8 μm pore size; Millipore, United States of America). The membranes were not coated with extracellular matrix. 16HBE cells were suspended at 2.5 × 10^5^ cells/mL, and 200 μL of serum-free medium was added to the upper chamber, while 600 μL RPMI-1640 medium containing 10% fetal bovine serum was placed in the lower chamber as a chemoattractant. After 24 h incubation at 37 °C in 5% CO_2_, non-migrated cells on the upper surface were gently removed with a cotton swab. Migrated cells on the lower surface were fixed with methanol for 30 min and stained with 0.1% crystal violet for 30 min. Cells were washed, air-dried, and counted in five randomly selected non-overlapping microscopic fields under an inverted microscope. The average number of migrated cells from the five fields was used to represent migration for each insert. Detailed experimental conditions are summarized in Supplementary Table S4.

#### 2.4.10 Western blot analysis

Total protein was extracted from cultured cells using RIPA lysis buffer supplemented with protease inhibitors. After centrifugation, protein concentrations were determined using a bicinchoninic acid (BCA) assay. Equal amounts of protein were separated by SDS–PAGE and transferred onto PVDF membranes. Membranes were blocked and incubated with primary antibodies against epithelial–mesenchymal transition (EMT)-related markers and GAPDH, followed by HRP-conjugated secondary antibodies. Protein bands were visualized using enhanced chemiluminescence and quantified with ImageJ software, using GAPDH as a loading control and background subtraction. Key experimental procedures and reagents are listed in Supplementary Tables S3 and S4.

### 2.5 Statistical analysis

Data were analyzed using GraphPad Prism 10.0. Continuous data are expressed as mean ± standard deviation (SD). Comparisons between two groups were performed using Student’s t-test, and among multiple groups using one-way ANOVA followed by Tukey’s post hoc multiple-comparison test. Semi-quantitative histological scores (e.g., HE and PAS scores) were analyzed using non-parametric tests: Mann–Whitney U test for two groups and Kruskal–Wallis test followed by Dunn’s multiple-comparison test for multiple groups. All tests were two-sided, and *p* < 0.05 was considered statistically significant. Significance levels were defined as follows: *p < 0.05, **p < 0.01, ***p < 0.001, ****p < 0.0001 and “ns” for non-significant differences. The value of n represents the number of independent biological replicates.

## 3 Results

### 3.1 Type 2 airway inflammation model establishment

**FIGURE 1:**
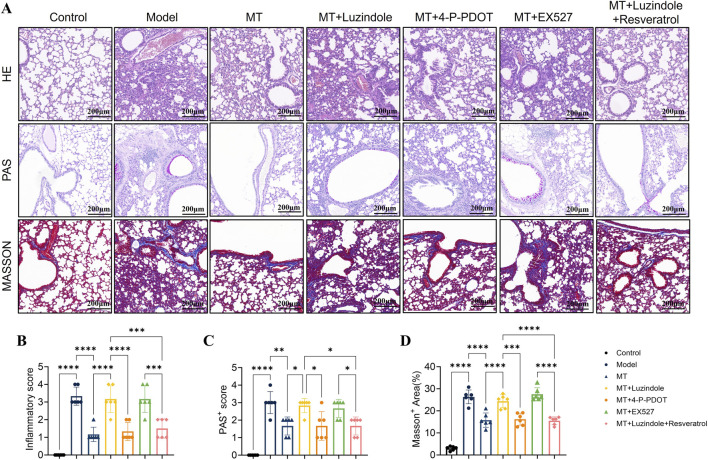
Melatonin attenuates lung inflammation and pathology in mice via MT1 signaling. **(A)** Representative lung sections stained with H&E, PAS, and Masson’s trichrome. **(B–D)** Quantification of inflammation score, PAS-positive score, and Masson-positive area (%) after Luzindole, Resveratrol, EX527 or combined treatment. n = 6; *p < 0.05, **p < 0.01, ***p < 0.001, ****p < 0.0001.

**FIGURE 2:**
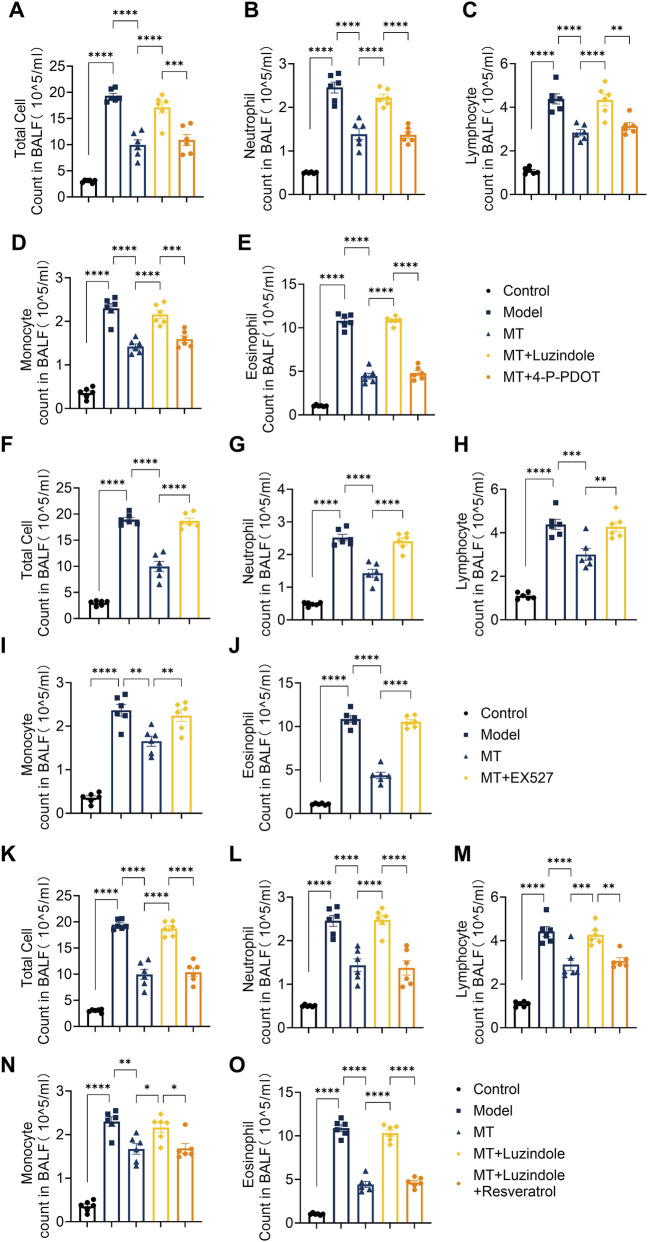
Melatonin reduces inflammatory cell infiltration in mouse lungs via the MT1-Sirt1 pathway. **(A–E)** Total cells, neutrophils, lymphocytes, monocytes and eosinophils in bronchoalveolar lavage fluid (BALF) following separate administration of Luzindole and Resveratrol. **(F–J)** Total cells, neutrophils, lymphocytes, monocytes and eosinophils in bronchoalveolar lavage fluid (BALF) after EX527 treatment. **(K–O)** Total cells, neutrophils, lymphocytes, monocytes and eosinophils in BALF after Luzindole or Luzindole combined with resveratrol treatment. n = 6; *p < 0.05, **p < 0.01, ***p < 0.001, ****p < 0.0001.

An ovalbumin (OVA)-induced murine model of type 2 airway inflammation was established as previously described (Wang et al., 2025). Compared with controls, OVA-challenged mice showed marked inflammatory cell infiltration around the airways and vessels (H&E), increased goblet cell hyperplasia and mucus secretion (PAS), and enhanced collagen deposition around the airway walls (Masson) (Figures 1A–D). In addition, bronchoalveolar lavage fluid (BALF) analysis revealed a significant increase in inflammatory cells, particularly eosinophils (Figures 2A–E), confirming successful establishment of the type 2 airway inflammation model.

### 3.2 Melatonin supplementation attenuates allergic airway inflammation

**FIGURE 3:**
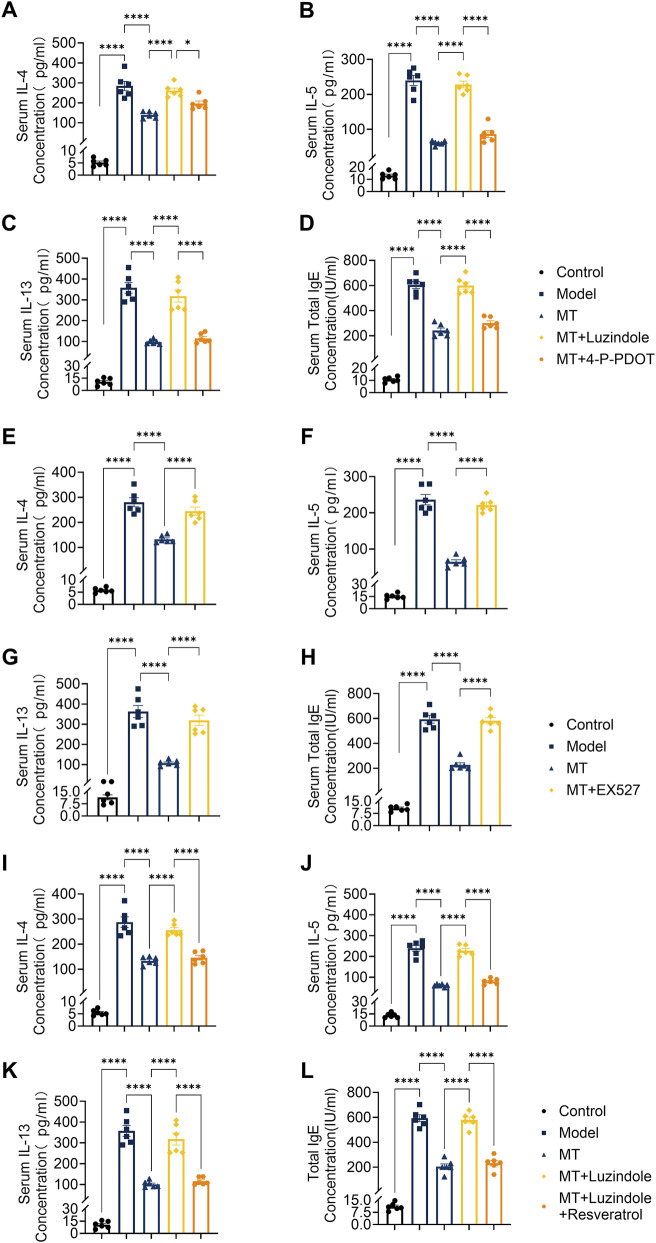
Melatonin attenuates Th2-type cytokine release and mucin deposition via the MT1-Sirt1 pathway. **(A–D)** Levels of IL-4, IL-5, IL-13, and IgE in serum after separate treatment with Luzindole and Resveratrol. **(E–H)** Levels of IL-4, IL-5, IL-13, and IgE in serum after EX527 treatment. **(I–L)** Levels of IL-4, IL-5, IL-13, and IgE in serum after Luzindole or Luzindole combined with resveratrol treatment. n = 6; *p < 0.05, ****p < 0.0001.

**FIGURE 4:**
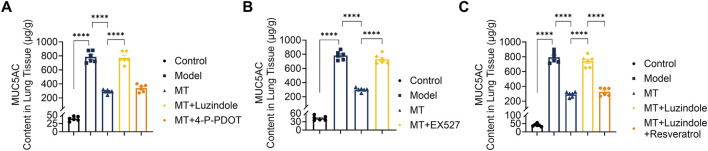
MUC5AC protein levels in lung tissues measured by ELISA. **(A)** Expression of MUC5AC in lung tissues after separate treatment with Luzindole and Resveratrol. **(B,C)** Expression of MUC5AC in lung tissues after EX527, Luzindole, or Luzindole combined with resveratrol treatment. n = 6; ****p < 0.0001.

To evaluate the therapeutic potential of exogenous melatonin against ovalbumin (OVA)-induced allergic airway inflammation, melatonin was administered as a pharmacological intervention. Histopathological analysis using H&E and PAS staining showed reduced inflammatory cell infiltration and mucus hypersecretion in melatonin-treated mice compared with the model group (Figures 1A,B,D). Consistently, inflammatory cell counts in bronchoalveolar lavage fluid (BALF) were significantly decreased (Figure 2), along with reduced serum levels of type 2 cytokines (IL-4, IL-5, IL-13) and immunoglobulin E (IgE) (Figure 3). Furthermore, MUC5AC expression in lung tissues was markedly reduced after melatonin treatment (Figure 4), indicating decreased mucus production. Collectively, these results demonstrate the protective effect of melatonin against type 2 airway inflammation.

### 3.3 MT1 receptor expression is preferentially altered in lung tissue

**FIGURE 5:**
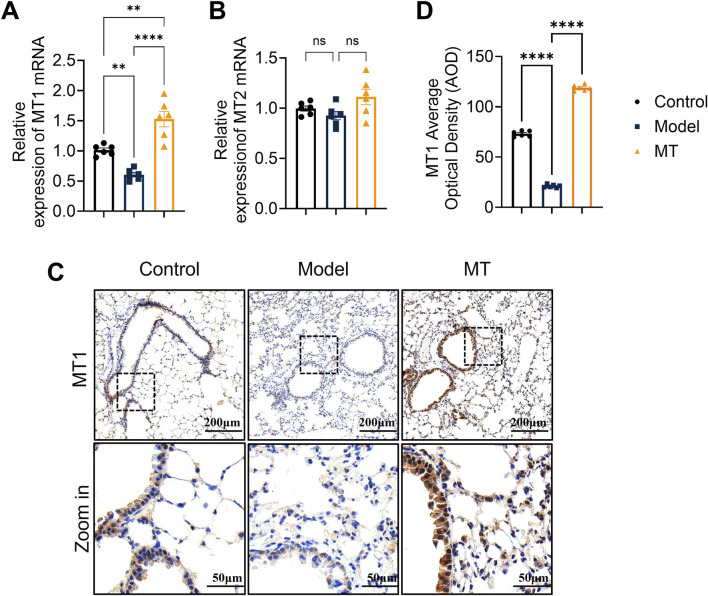
Melatonin exerts its effects in mouse lung tissues via the MT1 receptor. **(A,B)** qRT-PCR analysis of MT1 and MT2 mRNA expression in mouse lung tissues. **(C)** Immunohistochemical staining of MT1 in mouse lung tissues. **(D)** Immunohistochemical scoring of MT1. n = 6; ns, not significant; **p < 0.01, ****p < 0.0001.

To clarify how OVA and melatonin affect circadian regulation in mouse lung tissue, MT1 and MT2 receptor expression was analyzed. qRT-PCR showed that OVA significantly reduced MT1 mRNA levels, whereas MT2 expression was not significantly affected (Figures 5A,B). Melatonin restored MT1 expression but had no significant effect on MT2. Immunohistochemistry showed that MT1 was mainly localized in bronchial epithelial cells, where its expression was reduced by OVA and restored by melatonin (Figures 5C,D). These results indicate that MT1 is the receptor predominantly altered in this model and suggest a functional role for MT1 in mediating melatonin’s effects.

### 3.4 Melatonin regulates Sirt1 and downstream clock genes via MT1 receptor

**FIGURE 6:**
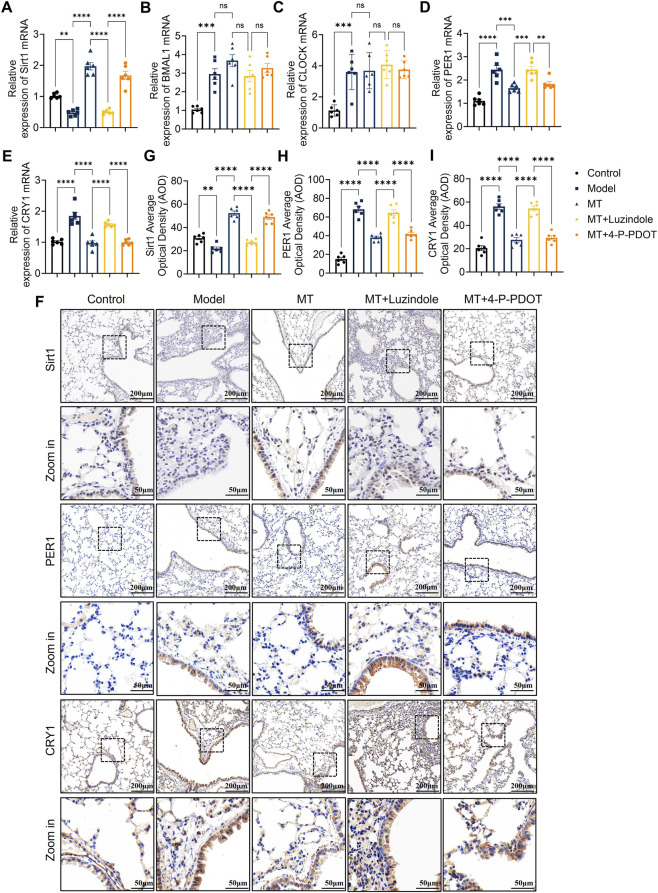
Melatonin regulates circadian gene expression via the MT1-Sirt1 pathway. **(A–E)** qRT-PCR analysis of BMAL1, CLOCK, PER1, CRY1 and Sirt1 mRNA in mouse lungs after Luzindole or 4-P-PDOT treatment. **(F–I)** Immunohistochemical staining and scoring of Sirt1, PER1 and CRY1 in lungs under the same treatments. n = 6; ns, not significant; **p < 0.01, ***p < 0.001, ****p < 0.0001.

To determine which melatonin receptor mediates the regulatory effects of melatonin, mice were treated with melatonin in the presence of either the nonselective melatonin receptor antagonist luzindole or the MT2-specific antagonist 4-P-PDOT. qRT-PCR analysis demonstrated that OVA challenge significantly reduced Sirt1 mRNA expression, which was reversed by melatonin treatment (Figure 6A). Similar expression patterns were observed for BMAL1, CLOCK, PER1, and CRY1 (Figures 6B–E). Importantly, the effects of melatonin were abolished by luzindole but not by 4-P-PDOT. Immunohistochemical staining further showed that these proteins were primarily localized in bronchial epithelial cells (Figures 6F–I). These results support MT1-dependent regulation of circadian genes by melatonin.

### 3.5 Melatonin attenuates pulmonary pathology and goblet cell hyperplasia via the MT1 receptor

To investigate the roles of MT1 and MT2 receptors in the protective effects of melatonin on airway remodeling and inflammation, histopathological analyses were performed following receptor blockade. H&E staining showed that OVA exposure induced severe airway inflammation, epithelial damage, and goblet cell hyperplasia (Figures 1A,B). Melatonin treatment markedly alleviated these pathological changes, an effect abolished by Luzindole and partially reversed by 4-P-PDOT (Figures 1A,B). Consistently, PAS staining demonstrated reduced goblet cell hyperplasia, ELISA revealed decreased pulmonary MUC5AC levels, and Masson’s trichrome staining showed attenuated airway fibrosis in the melatonin-treated group (Figures 1A,B,D, 4A). These findings indicate that MT1-mediated melatonin signaling suppresses mucus overproduction and collagen deposition, thereby contributing to the protective effects of melatonin on airway pathology.

### 3.6 Melatonin attenuates pulmonary inflammatory cell infiltration via MT1 receptor activation

To investigate the role of melatonin receptors in regulating pulmonary inflammation, total and differential cell counts were performed on BALF samples from each group. Melatonin significantly reduced total BALF cell counts in OVA-challenged mice, along with marked decreases in neutrophils, monocytes, lymphocytes, and eosinophils (Figure 2), consistent with previous findings. These effects were abolished by Luzindole, a non-selective MT1/MT2 antagonist, but not by the MT2-selective antagonist 4-P-PDOT (Figures 2A–E). This highlights the importance of MT1 activation in suppressing airway inflammation.

### 3.7 Melatonin attenuates Th2 cytokine release and regulates airway mucin expression via MT1 receptor signaling

Serum levels of Th2-associated cytokines IL-4, IL-5, IL-13, and IgE were significantly reduced in melatonin-treated OVA-induced mice (Figure 3). This inhibitory effect was abolished by the non-selective MT1/MT2 antagonist Luzindole but remained unaffected by the MT2-selective antagonist 4-P-PDOT (Figures 3A–D), indicating that melatonin suppresses Th2 cytokine production primarily through MT1 receptor activation. Consistent with the reduction in Th2 inflammation, melatonin also decreased lung MUC5AC expression (Figure 4A). This effect was reversed by Luzindole but not by 4-P-PDOT, suggesting that MT1 signaling mediates the inhibitory effect of melatonin on mucus production under type 2 inflammatory conditions.

### 3.8 Melatonin regulates clock gene expression via the MT1-Sirt1 pathway

**FIGURE 7:**
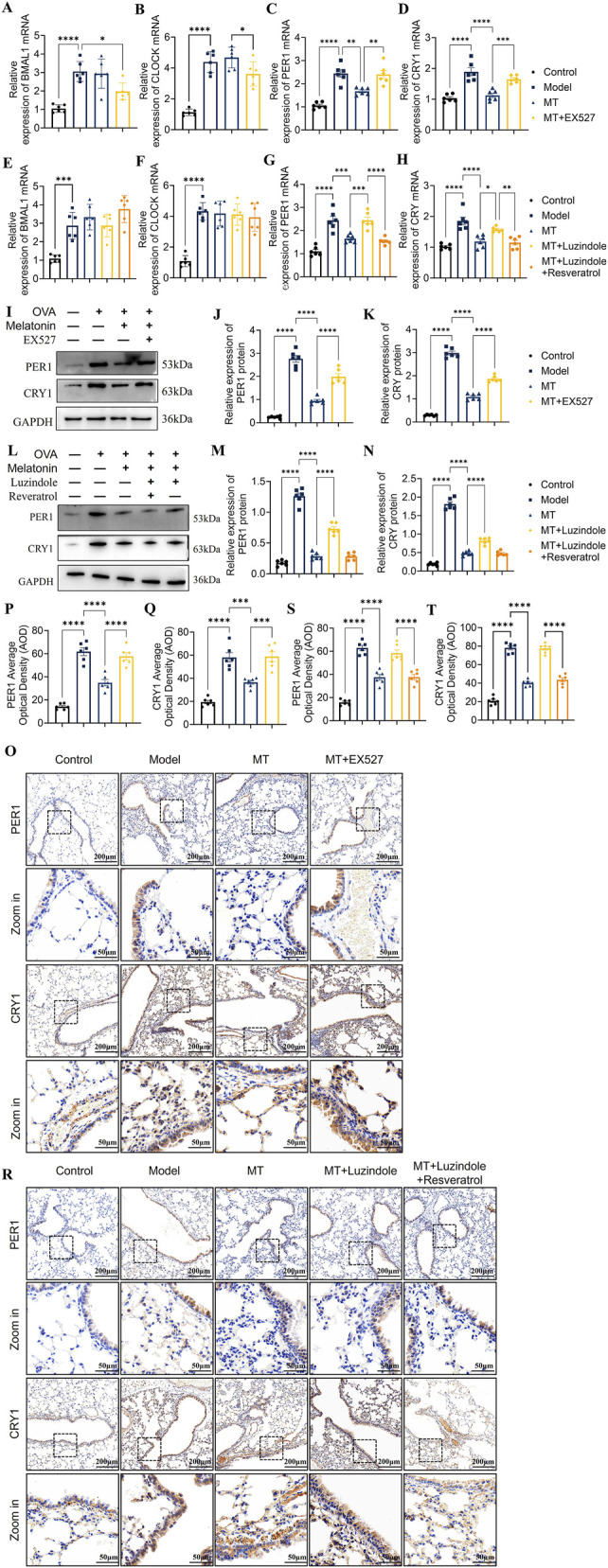
Melatonin regulates circadian gene expression in mouse lungs via the MT1-Sirt1 pathway **(A–D)** qRT-PCR analysis showing the effects of EX527 on BMAL1, CLOCK, PER1, and CRY1 mRNA expression. **(E–H)** qRT-PCR analysis showing that combined Luzindole and Resveratrol treatment modulates BMAL1, CLOCK, PER1, and CRY1 mRNA levels. **(I)** Western blot analysis of PER1 and CRY1 protein levels after EX527 treatment. **(J,K)** Quantification of PER1 and CRY1 protein expression. **(L)** Western blot analysis of PER1 and CRY1 protein levels after combined Luzindole and Resveratrol treatment. **(M,N)** Quantification of PER1 and CRY1 protein expression. **(O–Q)** Immunohistochemical staining and scoring of PER1 and CRY1 in lungs after EX527 treatment. **(R–T)** Immunohistochemical staining and scoring after combined Luzindole and Resveratrol treatment. n = 6; *p < 0.05, **p < 0.01, ***p < 0.001, ****p < 0.0001.

To investigate whether melatonin modulates downstream clock gene expression through the MT1-Sirt1 axis, OVA-induced type 2 airway inflammation mice were treated with the Sirt1 inhibitor EX527 alone, or co-treated with the melatonin receptor antagonist Luzindole and the Sirt1 agonist Resveratrol. qRT-PCR analysis showed that OVA and melatonin treatments affected BMAL1, CLOCK, PER1, and CRY1 mRNA levels consistent with previous results. Neither EX527, Luzindole, nor the combination of Luzindole and Resveratrol caused statistically significant changes in BMAL1 and CLOCK transcription compared to melatonin alone (Figures 6B–E; Figures 7A–H). However, EX-527 and Luzindole each increased PER1 and CRY1 mRNA expression (Figures 6D,E, 7C,D), while the combined treatment of Luzindole and Resveratrol significantly decreased PER1 and CRY1 transcription (Figures 7G,H). Protein analysis by Western blot revealed that PER1 and CRY1 levels paralleled mRNA expression: EX527 and Luzindole individually upregulated PER1 and CRY1 protein levels, whereas their combination with Resveratrol markedly suppressed these proteins (Figures 7I–N; Supplementary Figures S1, S2). Immunohistochemistry further confirmed these findings, showing increased PER1 and CRY1 expression in lung epithelial cells after EX527 or Luzindole treatment, but significant inhibition when Luzindole was combined with Resveratrol (Figures 7O–T). This finding links melatonin signaling to circadian regulation during airway inflammation.

### 3.9 Melatonin alleviates lung pathological injury, inflammatory cell infiltration, Th2 cytokine release, and mucin expression via the MT1-Sirt1 pathway

To further evaluate the role of Sirt1 in melatonin-mediated protection, OVA-induced mice were treated with the Sirt1 inhibitor EX-527 or co-treated with the melatonin receptor antagonist Luzindole and the Sirt1 agonist Resveratrol. Histological analyses (H&E, PAS, and Masson staining) showed that EX-527 largely reversed the protective effects of melatonin on lung inflammatory infiltration, mucus hypersecretion, and collagen deposition (Figures 1A–D, 2F,G, 4B). In contrast, activation of Sirt1 by Resveratrol partially alleviated airway inflammation and remodeling even under MT1 receptor blockade by Luzindole (Figures 1, 2K–O, 4C).

Consistently, EX-527 significantly increased total cell counts and inflammatory cell subsets including neutrophils, monocytes, lymphocytes, and eosinophils—in bronchoalveolar lavage fluid (BALF) (Figures 2F,G), whereas combined Luzindole and Resveratrol treatment reduced these cell counts despite MT1 inhibition (Figures 2K–O). Serum analysis further showed that EX-527 elevated Th2-associated cytokines IL-4, IL-5, IL-13, and IgE levels (Figures 3E–H), while Resveratrol reversed these increases under MT1 blockade (Figures 3I–L). Similarly, EX-527 increased lung tissue expression of the mucin protein MUC5AC, whereas combined Luzindole and Resveratrol treatment significantly reduced its expression (Figures 4B,C). Collectively, these findings highlight the functional importance of the MT1–Sirt1 pathway in regulating airway inflammation and remodeling.

### 3.10 Melatonin partially restores PER1 and CRY1 circadian-related expression disrupted by inflammation

**FIGURE 8:**
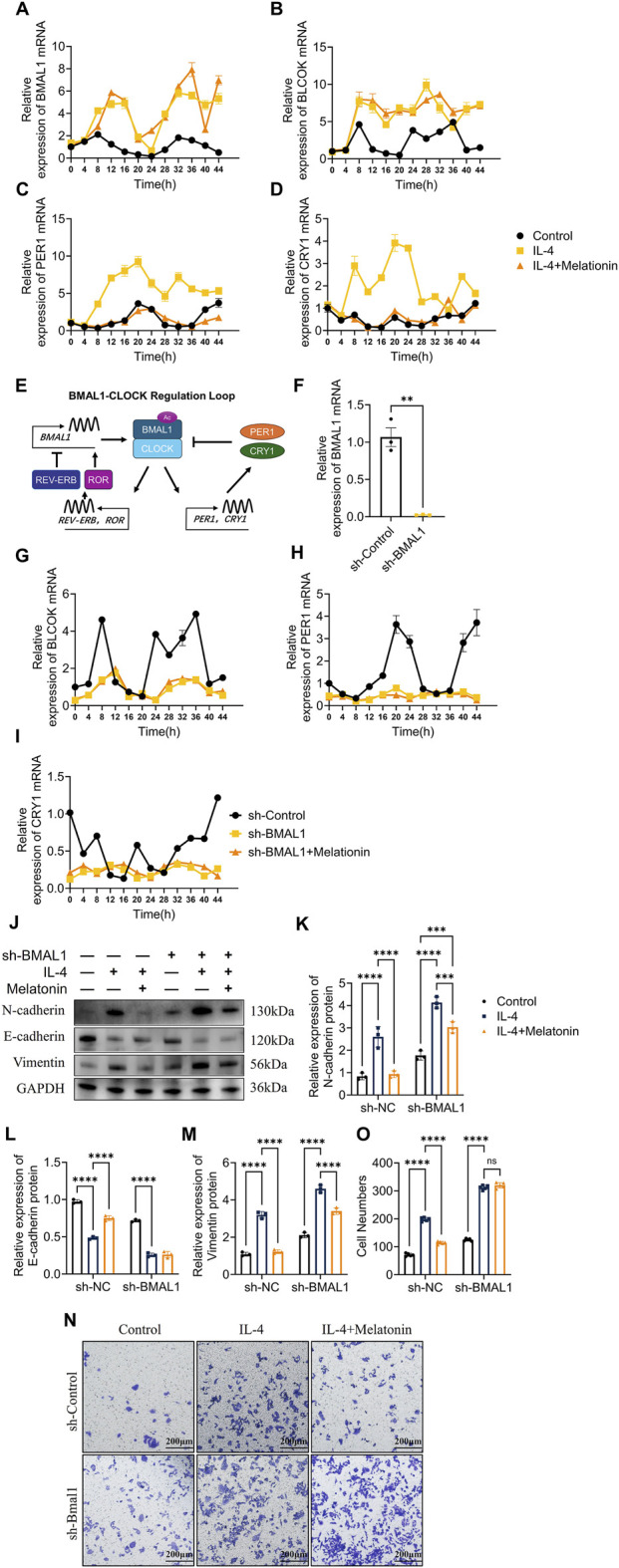
Melatonin regulates circadian genes to inhibit inflammation, EMT, mucin production, and cell migration in 16HBE cells **(A–D)** qRT-PCR showing BMAL1, CLOCK, PER1, and CRY1 mRNA in IL-4-treated 16HBE cells, with melatonin restoring circadian gene expression. **(E)** Schematic of the BMAL1/CLOCK-PER1/CRY1 negative feedback loop. **(F–I)** qRT-PCR showing circadian gene disruption in BMAL1 knockdown (sh-BMAL1) cells, with or without melatonin. **(J–M)** Western blot and quantification of EMT markers (N-cadherin, E-cadherin, Vimentin) indicating melatonin suppresses EMT via circadian gene modulation. n = 3; **p < 0.01,***p < 0.001. **(N,O)** Transwell migration assay and quantification showing reduced 16HBE cell migration after melatonin treatment. n = 5; ns, not significant; ****p < 0.0001.

We investigated the effects of inflammation on circadian clock gene expression patterns in 16HBE cells. IL-4 stimulation disrupted the altered the expression patterns of BMAL1, CLOCK, PER1, and CRY1, resulting in increased and dysregulated mRNA expression compared to control cells (Figures 8A–D). Melatonin treatment partially normalized the expression patterns of PER1 and CRY1 transcripts but did not markedly affect BMAL1 and CLOCK expression patterns. These findings suggest that type 2 airway inflammation disrupts circadian clock gene expression and that melatonin preferentially modulates PER1 and CRY1 expression under these conditions. To clarify the role of BMAL1 in this process, we generated BMAL1 knockdown (KD) 16HBE cells using shRNA lentiviral infection, achieving significant suppression of BMAL1 mRNA (Figure 8H). Serum shock–induced circadian gene expression changes of CLOCK, PER1, and CRY1 was substantially reduced in BMAL1-KD cells, and melatonin did not restore their expression patterns (Figures 8G–I). These results indicate that BMAL1 is required for melatonin-associated modulation of circadian clock gene expression under inflammatory conditions.

### 3.11 Melatonin inhibits EMT, mucin expression, and cell migration via clock gene regulation

Given the critical role of epithelial-mesenchymal transition (EMT) in airway remodeling, we evaluated IL-4 and melatonin effects on EMT markers in sh-Control and sh-BMAL1 cells. IL-4 induced EMT by increasing N-cadherin and Vimentin and decreasing E-cadherin expression, effects reversed by melatonin in control cells (Figures 8J–M; Supplementary Figure S3). BMAL1 knockdown promoted EMT marker expression; IL-4 further enhanced EMT, but melatonin failed to inhibit EMT in BMAL1-KD cells, demonstrating clock gene dependency. Transwell migration assays demonstrated that IL-4 significantly increased migration of 16HBE cells, an effect that was attenuated by melatonin in control cells. Silencing BMAL1 further enhanced cell migration, and IL-4 induced an additional increase; however, melatonin failed to reverse this effect in BMAL1-knockdown cells (Figures 8N,O). This supports a role for circadian regulation in the anti-remodeling effects of melatonin.

## 4 Discussion

Asthma is a chronic respiratory disorder driven by complex genetic and environmental interactions and characterized by airway inflammation, hyperresponsiveness, and structural remodeling (Venkatesan, 2023; Chen et al., 2025; Ravindran et al., 2025). Type 2 immune responses are central to disease development, promoting eosinophilic inflammation, excessive mucus production, and epithelial injury (Maun et al., 2019; Menzies-Gow et al., 2020; Mims, 2015). Although current treatments are effective for many patients, some patients still experience exacerbations, underscoring the need to better define the molecular mechanisms driving airway inflammation and to identify new therapeutic targets (Yang et al., 2025; Principe and Jarjour, 2025). Increasing evidence indicates that circadian rhythm disturbances contribute to immune imbalance and chronic inflammatory conditions, including asthma (Froy and Weintraub, 2025; Jiang et al., 2018; Naidu et al., 2010; Sutherland, 2005). Circadian clock genes regulate diverse physiological processes, such as immune cell movement, cytokine release, and epithelial barrier integrity (Chertoff et al., 2022; Wang et al., 2018; Li et al., 2025). This study seeks to fill this gap by examining the role of melatonin, a central circadian regulator, in modulating airway inflammation through circadian-associated signaling pathways.

In the present work, we show that melatonin alleviates airway inflammation by modulating the core circadian clock genes CRY1 and PER1 via an MT1 receptor-dependent, Sirt1-mediated signaling pathway. Experiments in both mouse models and airway epithelial cells revealed that melatonin suppressed inflammatory responses while concurrently reshaping circadian gene expression. These effects required intact MT1 signaling and were associated with enhanced Sirt1 expression and activity. Together, these findings indicate that melatonin exerts anti-inflammatory effects through coordinated regulation of circadian and immune pathways.

At the mechanistic level, our results indicate that melatonin-induced activation of Sirt1 is accompanied by reduced expression of PER1 and CRY1. Sirt1 is a NAD^+^-dependent deacetylase involved in immune regulation, oxidative stress control, and inflammatory processes. Previous studies have shown that Sirt1 activation negatively modulates inflammatory signaling pathways (Ross et al., 2022; Yang et al., 2024; Liu et al., 2019). In airway epithelial cells, Sirt1 has also been linked to the preservation of epithelial barrier function and inflammatory mediator release. In line with these observations, our data support a role for Sirt1 as a key mediator connecting melatonin signaling with circadian gene regulation, thereby contributing to the suppression of airway inflammation.

The reduction in PER1 and CRY1 expression following melatonin exposure indicates a functional link between Sirt1 activity and the circadian clock system. Clock genes play a key role in shaping immune responses by controlling the timing and intensity of inflammatory signaling. Disruption of PER and CRY family members has been associated with heightened inflammatory reactions and greater vulnerability to airway inflammation. Through regulation of these genes, melatonin may contribute to the restoration of circadian stability and prevent excessive activation of inflammatory pathways. This mechanism offers a plausible explanation for the anti-inflammatory effects observed in this study and highlights the importance of circadian regulation in maintaining immune homeostasis in the airway.

Notably, our findings identify the MT1 receptor as a key upstream regulator of melatonin’s actions. Although melatonin has been widely reported to exert anti-inflammatory and antioxidant effects, its receptor-specific role particularly that of MT1 in controlling Sirt1 activity and circadian gene expression in airway inflammation has not been well defined (Xi et al., 2020; Chen et al., 2020; Liu et al., 2021; Zhao et al., 2021). The present data demonstrate that MT1 signaling is essential for melatonin-driven activation of Sirt1 and subsequent modulation of circadian genes. This observation underscores the importance of receptor-dependent melatonin signaling and suggests that changes in MT1 expression or function may contribute to circadian dysregulation and immune imbalance in asthma. Therapeutically, selective targeting of MT1-associated pathways may provide a more precise strategy than systemic melatonin administration alone.

These findings align with growing evidence that circadian rhythm integrity is closely associated with asthma severity and disease control (Lu et al., 2025; Scheer et al., 2021). Clinical observations indicate that asthma symptoms and airway responsiveness follow marked daily fluctuations, often worsening during the night or early morning. Circadian disruption, whether driven by environmental influences or genetic changes in clock genes, has been linked to heightened airway inflammation and poorer clinical outcomes (Sato et al., 2021; Fagnano et al., 2011; Smolensky et al., 2007; Teppan et al., 2025). The present data suggest that melatonin’s regulation of circadian gene expression may be one mechanism through which circadian homeostasis modulates airway inflammation, offering a biological rationale for chronotherapy-based strategies in asthma treatment.

Circadian disruption may aggravate the pulmonary inflammatory microenvironment by disturbing the temporal regulation of immune cell trafficking, cytokine secretion, and epithelial barrier integrity (Scheer et al., 2021; Smolensky et al., 2007). Such dysregulation can lead to persistent inflammatory signaling and increased susceptibility to airway injury. However, current asthma therapies, including corticosteroids and biologics targeting type 2 cytokines, primarily suppress downstream inflammatory pathways and do not directly address circadian dysregulation. Consequently, these treatments may not fully restore physiological immune rhythmicity or prevent inflammation associated with circadian misalignment, highlighting the need for therapeutic strategies that integrate circadian regulation with anti-inflammatory interventions.

In addition to its anti-inflammatory actions, our study shows that melatonin inhibits epithelial-mesenchymal transition (EMT) in airway epithelial cells. EMT is increasingly recognized as an important driver of airway remodeling, a defining feature of chronic and severe asthma (Feng et al., 2022). During EMT, epithelial cells adopt mesenchymal traits, resulting in increased migratory capacity, extracellular matrix deposition, and impaired barrier integrity (Sun et al., 2025). The suppression of EMT by melatonin therefore suggests that its protective effects extend beyond inflammation modulation to the maintenance of epithelial structure and function.

The capacity of melatonin to regulate EMT may be especially important in chronic airway diseases, where recurrent inflammation drives ongoing structural alterations. Prior studies have shown that melatonin can affect cellular plasticity and fibrotic responses in multiple tissues, including the lung (Pivonello et al., 2022; Gupta, 2023). In line with these reports, our results indicate that melatonin may attenuate airway remodeling by concurrently inhibiting inflammatory signaling and EMT-related processes. This combined effect could strengthen its therapeutic relevance in asthma, a condition in which inflammation and remodeling are tightly linked.

Several limitations should be considered. First, the findings are derived mainly from preclinical models, which may not fully reflect the heterogeneity of asthma in humans. Second, the absence of validation using patient-derived samples limits evaluation of the clinical relevance of the identified signaling pathways. Third, this study primarily examined acute inflammatory responses, and the impact of melatonin on chronic airway inflammation and long-term airway remodeling requires further investigation.

Despite these limitations, this study provides mechanistic support for a previously underrecognized role of melatonin in linking circadian regulation with airway inflammation. The identification of an MT1-Sirt1-circadian gene axis offers new insight into how circadian signals interact with immune pathways in the airway. Future studies should evaluate long-term effects of melatonin and explore the therapeutic potential of targeting MT1-dependent circadian signaling in type 2 airway diseases.

## 5 Conclusion

In conclusion, this study supports a mechanism whereby melatonin attenuates type 2 airway inflammation through MT1-dependent activation of Sirt1 and subsequent modulation of circadian rhythm genes. These findings deepen our understanding of the interplay between circadian biology and airway inflammation and provide a theoretical basis for the development of melatonin-based or circadian-targeted therapeutic strategies for asthma and related respiratory diseases. While prior studies have reported effects of melatonin on inflammation and circadian regulation, our results further implicate the MT1-Sirt1-circadian gene axis in the context of type 2 airway inflammation.

## Data Availability

The original contributions presented in the study are included in the article/Supplementary Material, further inquiries can be directed to the corresponding authors.
