# Supplementary material for: Melatonin: a promising therapy to combat type 2 airway inflammation via MT1-Sirt1 pathway
Source: Front Pharmacol. 2026 Apr 17;17:1805178. doi: 10.3389/fphar.2026.1805178 (PMC13133056; doi:10.3389/fphar.2026.1805178)
Supplement: Supplementary file 1 [file Table1.docx]

Table S1 Primer sequences used for qRT-PCR in mouse lung tissues

| Gene primer sequence | | |
| --- | --- | --- |
| mRNA | Primers | Sequences (5′-3′) |
| mGAPDH | Forward | AGGTCGGTGTGAACGGATTTG |
|  | Reverse | GGGGTCGTTGATGGCAACA |
| mMT1 | Forward | AAGAGTGAGTTGGGACACCTT |
|  | Reverse | CGAGACAATACAATGGCCTCC |
| mMT2 | Forward | GCCTGCAAATGCAAACAATGC |
|  | Reverse | AGCTGCACTTGTCGGAAGC |
| mSIRT1 | Forward | TGATTGGCACCGATCCTCG |
|  | Reverse | CCACAGCGTCATATCATCCAG |
| mBMAL1 | Forward | ACAGTCAGATTGAAAAGAGGCG |
|  | Reverse | GCCATCCTTAGCACGGTGAG |
| mCLOCK | Forward | AGGCTATTTGCCATTTGAAGTCT |
|  | Reverse | GCTCGTGACATTTTGCCAGATTT |
| mCRY1 | Forward | TTGCCTGTTTCCTGACTCGT |
|  | Reverse | GACAGCCACATCCAACTTCC |
| mPER1 | Forward | GAAAGAAACCTCTGGCTGTTCCT |
|  | Reverse | GCTGACGACGGATCTTTCTTG |
| mMUC5AC | Forward | CAGGACTCTCTGAAATCGTACCA |
|  | Reverse | GAAGGCTCGTACCACAGGG |

Table S2 Primer sequences used for qRT-PCR in cell experiments

| Gene primer sequence | | |
| --- | --- | --- |
| mRNA | Primers | Sequences (5′-3′) |
| hGAPDH | Forward | GATTCCACCCATGGCAAATTC |
|  | Reverse | CTGGAAGATGGTGATGGGATT |
| hBMAL1 | Forward | GTACCAACATGCAACGCAAT |
|  | Reverse | CTCGGTCACATCCTACGACA |
| hCLOCK | Forward | GATGGGTTGGTGGAAGAAGA |
|  | Reverse | AGGTTTCCAGTCCTGTCGAA |
| hPER1 | Forward | CCAGCACCACTAAGCGTAAA |
|  | Reverse | CACCACACTCTCCGCCTTAT |
| hCRY1 | Forward | GGAGGGTTGGATTCATCATC |
|  | Reverse | CAACAGGGCAATAGCAGTGA |
| hMUC5AC | Forward | CTGTGAAGGTGGCTGACCAAGA3 |
|  | Reverse | AAGGTGTAGTAGGTGCCGTCGAA |

Table S3 List of reagents, antibodies, and kits used in this study

| Reagent Name | Manufacturer | Catalog Number |
| --- | --- | --- |
| Anhydrous Ethanol | Sinopharm Chemical Reagent Co., Ltd. | 100092683 |
| Xylene | Sinopharm Chemical Reagent Co., Ltd. | 10023418 |
| Environmentally Friendly Dewaxing Solution | Servicebio | G1128 |
| Universal Tissue Fixative | Servicebio | G1101 |
| Masson Stain Kit | Servicebio | G1006 |
| Differentiation Solution | Servicebio | G1039 |
| Neutral Balsam | Sinopharm Chemical Reagent Co., Ltd. | 10004160 |
| Anhydrous Ethanol | Sinopharm Chemical Reagent Co., Ltd. | 100092683 |
| Environmentally Friendly Dewaxing and Clearing Solution | Wuhan Servicebio Technology Co., Ltd. | G1128 |
| Xylene | Sinopharm Chemical Reagent Co., Ltd. | 10023418 |
| n-Butanol | Sinopharm Chemical Reagent Co., Ltd. | 100052190 |
| Hydrochloric Acid | Sinopharm Chemical Reagent Co., Ltd. | 10011028 |
| 20× Citrate Antigen Retrieval Solution (pH 6.0) | Wuhan Servicebio Technology Co., Ltd. | G1202 |
| 20× Tris-EDTA Antigen Retrieval Solution (pH 9.0) | Wuhan Servicebio Technology Co., Ltd. | G1203 |
| 20× Tris-EDTA Antigen Retrieval Solution (pH 8.0) | Wuhan Servicebio Technology Co., Ltd. | G1206 |
| PBS Buffer | Wuhan Servicebio Technology Co., Ltd. | G0002 |
| Continued Table |  |  |
| Universal Tissue Fixative (Neutral) | Wuhan Servicebio Technology Co., Ltd. | G1101 |
| 3% Hydrogen Peroxide Disinfectant | Anjie Gaoke |  |
| Bovine Serum Albumin (BSA) | Wuhan Servicebio Technology Co., Ltd. | GC305010 |
| Normal Rabbit Serum (Concentrated) | Wuhan Servicebio Technology Co., Ltd. | G1209 |
| Hematoxylin Stain Solution | Wuhan Servicebio Technology Co., Ltd. | G1004 |
| Hematoxylin Differentiation Solution | Wuhan Servicebio Technology Co., Ltd. | G1039 |
| Hematoxylin Bluing Solution | Wuhan Servicebio Technology Co., Ltd. | G1040 |
| Super Fast Drying Mounting Medium | Wuhan Servicebio Technology Co., Ltd. | G1404-100mL |
| Immunohistochemistry DAB Kit | Wuhan Servicebio Technology Co., Ltd. | G1212 |
| Sirt1 | Proteintech Group, Inc. | 60303-1-Ig |
| PER1 | Proteintech Group, Inc. | 13463-1-AP |
| CRY1 | Proteintech Group, Inc. | 13474-1-AP |
| GAPDH | Proteintech Group, Inc. | 60004-1-Ig |
| RIPA Lysis Buffer | Shanghai Beyotime Biotechnology Co., Ltd. | P0013C |
| PMSF | Shanghai Beyotime Biotechnology Co., Ltd. | ST506 |
| Continued Table |  |  |
| BCA Protein Assay Kit | Shanghai Beyotime Biotechnology Co., Ltd. | P0011 |
| SDS-PAGE Protein Loading Buffer | Shanghai Beyotime Biotechnology Co., Ltd. | P0015L |
| 10% SDS-PAGE Gel One-Step Ultra Rapid Preparation Kit | Shanghai Beyotime Biotechnology Co., Ltd. | P0917S |
| Horseradish Peroxidase-Labeled Goat Anti-Mouse IgG (H+L) | Shanghai Beyotime Biotechnology Co., Ltd. | A0216 |
| Horseradish Peroxidase-Labeled Goat Anti-Rabbit IgG (H+L) | Shanghai Beyotime Biotechnology Co., Ltd. | A0208 |
| BeyoECL Star (Enhanced Chemiluminescent Substrate) | Shanghai Beyotime Biotechnology Co., Ltd. | P0018AM |
| BeyoWB™ PVDF Membrane | Shanghai Beyotime Biotechnology Co., Ltd. | P0965 |
| PVDF Membrane Activation/Equilibration Solution | Shanghai Beyotime Biotechnology Co., Ltd. | P0021S |
| SDS-PAGE Running Buffer | Shanghai Beyotime Biotechnology Co., Ltd. | P0014D |
| Western Rapid Transfer Buffer | Shanghai Beyotime Biotechnology Co., Ltd. | P0572 |
| Western Blocking Buffer | Shanghai Beyotime Biotechnology Co., Ltd. | P0023B |
| Mouse IL-5 ELISA Kit | Shanghai Beyotime Biotechnology Co., Ltd. | PI620 |
| Continued Table |  |  |
| Mouse IL-4 ELISA Kit | Shanghai Beyotime Biotechnology Co., Ltd. | PI612 |
| Mouse IL-13 ELISA Kit | Shanghai Beyotime Biotechnology Co., Ltd. | PI539 |
| Mouse IgE ELISA Kit | Shanghai Beyotime Biotechnology Co., Ltd. | PI476 |
| Mouse MUC5AC ELISA Kit | Wuhan Saipei Biotechnology Co., Ltd. | SP13899 |
| FastPure Cell/Tissue Total RNA Isolation Kit | Nanjing Vazyme Biotech Co., Ltd. | RC112 |
| HiScript III 1st Strand cDNA Synthesis Kit | Nanjing Vazyme Biotech Co., Ltd. | RC312 |
| Taq Pro Universal SYBR qPCR Master Mix | Nanjing Vazyme Biotech Co., Ltd. | Q712 |
| Dexamethasone | MedChemExpress | HY-14648 |
| Methacholine chloride | MedChemExpress | HY-A0083 |
| Ovalbumin (OVA) | Sangon Biotech (Shanghai) Co., Ltd. | A003049 |
| 4-P-PDOT | MedChemExpress | HY-100609 |
| Luzindole | MedChemExpress | HY-101254 |
| Resveratrol | MedChemExpress | HY-16561 |
| EX-527 | MedChemExpress | HY-15452 |
| MT1 antibody | Absin | abs119628 |
| MT2 antibody | Absin | abs120311 |

Table S4 Summary of mouse lung tissue and cell experimental procedures

| Experiment | Key Steps / Conditions | Reagents / Equipment | Notes / Critical Points |
| --- | --- | --- | --- |
| Western blot | Tissue lysis: 80 mg/mL RIPA + 1 mM PMSF, ice; sonication 15-20 s cycles | Ultrasonic homogenizer, centrifuge | 4℃ rotation 30 min, 12,000 rpm 20 min; supernatant for protein quantification; include no-primary and positive/negative tissue controls |
|  | Protein quantification: BCA assay | BCA reagent A/B, microplate reader | Standard curve 0-1 mg/mL; normalize samples, add 6×Loading buffer, 100℃ 5 min, ice cool |
|  | SDS-PAGE & transfer | PVDF membrane, gel electrophoresis system | Electrophoresis 80-120 V; transfer 280 mA 80 min; block 120 min; primary antibody 1:1000 4℃ overnight; secondary antibody 1:10,000 37℃ 2 h |
| Paraffin embedding | Fix 4% PFA 24 h; gradient ethanol dehydration; xylene clearing; paraffin infiltration overnight; cut 4 μm sections | Microtome, water bath, oven | Sections baked overnight at 60℃; store at 4℃ |
| H&E staining | Deparaffinize & rehydrate; hematoxylin 5±1 min; wash; eosin 20 s; dehydration; mount | Ethanol series, hematoxylin, eosin, neutral resin | Control staining time to avoid tissue damage; gentle washing |
| PAS staining | Deparaffinize & rehydrate; oxidize 0.5-1% periodic acid 5-10 min; Schiff reagent 10–20 min; hematoxylin 2-4 min; dehydration; mount | Periodic acid, Schiff reagent, hematoxylin | Summer 10 min / winter 20 min Schiff incubation; wash until background is colorless |
| Continued Table |  |  |  |
| Masson’s trichrome | Deparaffinize & rehydrate; hematoxylin 5 min; acid ethanol differentiation 2 s; phloxine/red 5-10 min; phosphomolybdic acid 1-2 min; aniline blue 1-2 min; dehydration; mount | Hematoxylin, phloxine, phosphomolybdic acid, aniline blue | Monitor under microscope to control collagen/muscle color |
| Immunohistochemistry (IHC) | Deparaffinize & rehydrate; antigen retrieval: citrate buffer 0.01 M, high-pressure cooker (4 min 2200 W + 3 min 1000 W); H2O2 3% 10 min; block 10% BSA 1 h; primary antibody 4℃ overnight; secondary 37℃ 30 min; DAB 1 min; hematoxylin counterstain; dehydration; mount | Citrate buffer, HRP/DAB kit (ZLI-9028/9018), PBS, BSA | Maintain humidity; avoid over-staining; gentle washing; observe DAB development; include no-primary, isotype, positive/negative tissue controls |
| Transwell cell migration | Seed 2.5×10^5 cells/mL 16HBE cells in upper chamber (200 μL, serum-free); lower chamber 600 μL RPMI-1640+10% FBS; incubate 37℃, 5% CO2, 24 h; fix with methanol 1 mL 30 min; stain 0.1% crystal violet 30 min; wash & air dry; count migrated cells in 5 random non-overlapping fields | Transwell 8um (Millipore), inverted microscope | Avoid bubble formation; gentle removal of non-migrated cells; random field counting; include negative controls if applicable |
| Viral packaging | 293T cells 65-75% confluent; transfection with pLKO.1+ psPAX2+pMD2.G (DNA ratio 4:3:1) using PEI in Opti-MEM; 8 h post-transfection, replace with DMEM + 10% FBS; collect viral supernatant 48 h, filter 0.45um, store -80℃ | 293T cells, PEI, Opti-MEM, DMEM, 100mm dish | Maintain sterile conditions; gentle mixing of DNA-PEI complex; filter to remove debris |
| Continued Table |  |  |  |
| Viral infection of 16HBE cells | Seed 2×10^5 cells/well in 6-well plates, 45-55% confluence at infection; mix virus:serum-free 1640 1:1+polybrene 10 μg/mL; incubate 6 h; replace medium with 10% FBS 1640; puromycin selection 1 μg/mL for 3-5 days; validate knockdown by qRT-PCR | 16HBE cells, polybrene, puromycin | Include uninfected negative control; monitor cell viability; maintain 37°C, 5% CO2 |
| Serum shock for circadian synchronization | Seed 1×10⁶ cells/well in 6-well plates; culture in serum-free RPMI-1640 for 24 h; treat with 50% horse serum in RPMI-1640 for 2 h; replace with serum-free medium (ZT0); collect cells every 4 h from ZT0-ZT44. At each time point, medium was aspirated, cells were washed with PBS, and lysed directly on the plate for RNA extraction. | 16HBE cells, horse serum, 1640 medium | T0 was defined as the time of serum shock washout; subsequent ZT values represent hours post-synchronization in vitro. Consistent timing was used for all sampling points. |
| RNA extraction & qRT-PCR | Extract total RNA from cells using FastPure Cell/Tissue RNA Isolation Kit; synthesize cDNA; perform qRT-PCR | FastPure RNA kit (Vazyme, RC112), qPCR system | Follow manufacturer’s protocol; include no-template controls |

Note: All experiments included appropriate controls, such as no-primary, isotype, positive and negative/KO samples, as detailed in each experimental entry.
